# An automated and reproducible workflow for running and analyzing neural simulations using Lancet and IPython Notebook

**DOI:** 10.3389/fninf.2013.00044

**Published:** 2013-12-30

**Authors:** Jean-Luc R. Stevens, Marco Elver, James A. Bednar

**Affiliations:** ^1^School of Informatics, Institute for Adaptive and Neural Computation, University of EdinburghEdinburgh, UK; ^2^School of Informatics, Institute for Computing Systems Architecture, University of EdinburghEdinburgh, UK

**Keywords:** IPython, pandas, reproducibility, workflow, simulation, batch computation, provenance, big data

## Abstract

Lancet is a new, simulator-independent Python utility for succinctly specifying, launching, and collating results from large batches of interrelated computationally demanding program runs. This paper demonstrates how to combine Lancet with IPython Notebook to provide a flexible, lightweight, and agile workflow for fully reproducible scientific research. This informal and pragmatic approach uses IPython Notebook to capture the steps in a scientific computation as it is gradually automated and made ready for publication, without mandating the use of any separate application that can constrain scientific exploration and innovation. The resulting notebook concisely records each step involved in even very complex computational processes that led to a particular figure or numerical result, allowing the complete chain of events to be replicated automatically. Lancet was originally designed to help solve problems in computational neuroscience, such as analyzing the sensitivity of a complex simulation to various parameters, or collecting the results from multiple runs with different random starting points. However, because it is never possible to know in advance what tools might be required in future tasks, Lancet has been designed to be completely general, supporting any type of program as long as it can be launched as a process and can return output in the form of files. For instance, Lancet is also heavily used by one of the authors in a separate research group for launching batches of microprocessor simulations. This general design will allow Lancet to continue supporting a given research project even as the underlying approaches and tools change.

## 1. Introduction

Computational neuroscience is a rapidly developing scientific field that relies on a large ecosystem of software tools that is continually evolving as high-performance computing infrastructure is updated. Every computational neuroscientist must therefore keep up with new developments in neuroscience, software engineering, and computer hardware while advancing novel computational theories of the nervous system. The drive to explore different scientific hypotheses rapidly has made Python the language of choice for many researchers due to its flexibility and wide range of libraries already provided. Despite this fast pace of change, it is crucial that results remain reproducible once they are obtained, if computational neuroscientists are to have long-term confidence in the integrity of their work.

The formidable challenges associated with developing replicable scientific publications in a rapidly advancing field are well recognized by the computational neuroscience community. The difficulties include problems replicating results between simulators (Crook et al.,^2013^) and insufficiently constrained model parameters in publications (Nordlie et al., 2009), along with an important debate about the distinction between replicability and reproducibility (Drummond, 2009; Freire et al., 2011). Fundamentally, neuroscience is concerned with the study of dynamic, history dependent biological systems of exceedingly high dimensionality. Although computational models abstract away most of the complexity of nervous systems by necessity, it is still a formidable challenge to communicate this type of work to other scientists while also capturing the key properties of the biological system under study. These broad issues must be addressed by the community as a whole, and cannot be solved by any one piece of software.

The approach we present to improve reproducibility is by offering a small number of useful utilities that first aim to improve a researcher's scientific productivity. If properly designed and useful enough to become a core part of a researcher's regular workflow, it is hoped that such tools will allow reproducible science to emerge naturally as researchers seek to increase productivity. This approach is in sharp contrast to more heavyweight automated scientific workflow systems (Curcin and Ghanem, 2008; Freire et al., 2014) that can be effective for mature research areas but would be constraining for this young and ever-changing field.

We developed the Lancet package as a small set of flexible, lightweight components that allow a researcher to generate and analyze large data sets more efficiently. These components are designed to help improve research efficiency by allowing the user to capture the essence of a scientific task with very little code and by catching errors early on, before expensive computational processes begin. By distilling a problem into a small number of short, declarative specifications, the researcher can focus on important scientific details, spending less time worrying about issues of implementation. Every component in Lancet is written to satisfy an immediate need; the end goal of generating automated, reproducible results should then be satisfied as a natural outcome of a clean and efficient solution to a problem.

By design, Lancet is a general utility, allowing it to work with any external tool or simulator. This ensures that as tools change or as researchers switch between software and platforms, the code written with Lancet remains unchanged. This generality is strictly enforced by the requirements of one of the authors, who is successfully applying Lancet outside the domain of computational neuroscience, i.e., to run simulations of varying microprocessor architectures. Lancet is pure, platform-independent Python with minimal dependencies, and supports both Python 2 and Python 3. Together, these properties should help ensure that code written using this utility will remain viable for the foreseeable future.

The goal of this new package is to allow reproducible, agile workflows to develop organically when used together with other tools, namely a suitable version control system and IPython Notebook. Since version 0.12 of IPython (Pérez and Granger, 2007), a notebook feature has been provided which allows code, data, and figures to be interactively explored while maintaining a complete record of the source code. Lancet is designed to integrate well with IPython and the pandas library (pandas.pydata.org), without having either of these two projects as a core dependency.

The next section introduces the components of Lancet, starting with a very small toy example of a workflow that begins with an initial specification and ends in a simple analysis. Section 3 provides an overview of the three main types of components offered in Lancet. At every stage, we show how these components make research tasks easier to complete by making the intentions of exploratory and publication-specific code clearer and more succinct. With the basic design established, Section 4 presents the full reproducible workflow, showing how Lancet can help turn reproducible science into practical reality when used together with IPython Notebook and other popular tools such as Git and the pandas data analysis library. To demonstrate that this workflow is both practical and relevant to a real research project, we then briefly describe how it was used to generate all the results in Stevens et al. (2013), recently published in the Journal of Neuroscience.

## 2. Basic lancet example

Python is a flexible, interpreted language that comes with many modules that extend the functionality of the base language. Closely related modules are collected into packages, some of which are included together with Python in the standard library and others that are available as third party libraries. The new Lancet package is designed to work together with the many excellent Python packages already available for scientific computing, to help capture and simplify a researcher's workflow. Lancet integrates particularly well with the interactive IPython notebook environment, which improves on Python's facilities for exploratory research and works across multiple platforms (Linux, MacOS, Windows). More information about Lancet, including installation instructions, may be found on Lancet's website (http://ioam.github.io/lancet).

**Figure 1:**
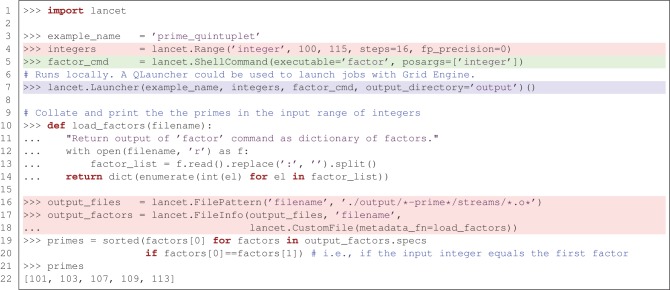
**A simple, end-to-end workflow using Lancet to factorize a range of integers, highlighted using the three colors used in the bullet points at the start of Section 2.** This simple example factorizes a list of integers with the factor command, with no other dependencies. The five prime numbers found are an example of a prime quintuplet, the closest admissible constellation of five consecutive prime numbers.

To introduce Lancet, we will first look at a minimal, toy example of a Python-based workflow with Lancet, listed in Figure 1. This example uses the simple factor command (included in GNU coreutils) to find the prime numbers that lie within a specific range of integers. Although brief, this example demonstrates how to use an initial specification of a parameter space to obtain results collated across 16 independent jobs. Section 4 will show how this approach fits into an agile, exploratory workflow. Meanwhile, even this simple example illustrates some of the key component types that are commonly applicable to many research tasks:

- *What you aim to achieve*. It is common to define a parameter space to be explored by some simulator or analysis tool. In Figure 1 this is the list of integers to factorize, highlighted in red. This level of specification expresses the scientific goal and is normally both *tool-independent* and *platform-independent*. Given a parameter space, it is conceivable that the desired results may be achieved using alternative software tools executed on different platforms. When exploring a parameter space, the key information is specified by the set of parameters explored and not by the details of the software used.
- *How you intend to achieve your goal*. This refers to the target software that runs a model or performs an analysis. In Figure 1 this is the factor command which factorizes integers, as highlighted in green. This type of specification is often *platform-independent* but *tool-dependent*, encapsulating how a specific piece of software is to be invoked with tool-dependent arguments, independent of the computational platform on which the software is run.
- *Where you want to execute the task*. If the software can run on multiple different platforms, there may be alternative ways to execute the tool. Executing a task in a particular environment is normally *platform-dependent* but *tool-independent*. In Figure 1 the factor command is executed locally using the Launcher class supplied by Lancet, highlighted in blue. By switching to the QLauncher class, the exact same task could be executed in parallel on a Grid Engine cluster without changing the rest of the code.

Of course, it is difficult to appreciate the advantages of using Lancet, if one simply wants to factor 16 small integers in Python. These advantages would be much more apparent if a multidimensional parameter space were to be explored with a complex neural simulator, as described below. Even so, non-Lancet Python code for launching these simple factor runs is likely to be longer, more error-prone and harder to read. Iteration over the input parameter space and output files (highlighted in red) would probably be expressed as multiple for loops, losing the flat structure of the example. Specification of the simulator (highlighted in green) and the code needed to execute it (highlighted in blue) would be interleaved and complex calls to the subprocess module would be required to execute jobs. Switching from local execution to Grid Engine would no longer be trivial.

This example demonstrates how Lancet can help free the researcher from such implementation details. Substantial code would also be needed to reproduce the way Lancet keeps your output files consistently organized (within timestamped folders by default) with a common directory structure, whether working locally or on a cluster. After executing the listing in Figure 1, a .info file will be generated together with the output, recording which Python version was used, the operating system on which the jobs were run, and the version of Lancet, alongside other useful metadata. Other information supplied by the user, such as the task description, versions of libraries and executables used, and other comments may be easily passed down to the metadata field of the .info file for storage. Lancet also offers a simple function that helps record version control information and improves reproducibility by maintaining an explicit log of all the parameters used. As shown later in Figure 5, all of this can be expressed clearly, succinctly, and declaratively, even for realistically complex sets of simulations.

## 3. Using lancet to rapidly specify a task

The example in Figure 1 briefly introduced the three core class hierarchies in Lancet. In this section, each of the three types is examined in greater detail, before in the next section we consider how Lancet can assist the natural development of an agile, reproducible workflow with IPython Notebook. A list of all the components available to the user, split into the three class families, is shown in Table 1.

**Table 1:**
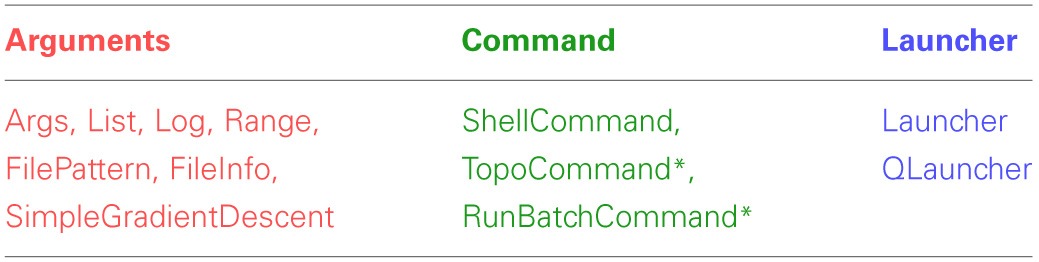
**Lancet components available for specifying jobs**.

All Arguments are subclasses of Args and specify static sets of parameters, except for SimpleGradientDescent which is an example of dynamic parameter optimization. ShellCommand is generic and included with Lancet whereas the Command classes marked by an asterisk are included with the Topographica simulator; other tools may offer their own custom Command classes. The Launcher class runs jobs locally, but other options are easy to implement, such as the QLauncher class for use on clusters.

First, Arguments declaratively specify the parameter space to be covered by a set of runs (see e.g., the Range object at the top of Figure 1, highlighted in red), or specify filenames and data of interest on the filesystem. The latter object type allows data on disk to be collated for analysis in Python, or for launching the next stage of a pipeline workflow.

Next, a Command class handles the interface to an external tool, allowing the rest of Lancet to remain simulator-independent. The example shown in Figure 1 uses a ShellCommand, which is supplied with Lancet for basic support of command-line programs. For supporting complex tools and simulators, Command can be subclassed while reimplementing only a constructor and a call method. As a workflow develops over time, it is likely that a user will want to make a custom Command to allow full control over important tools being used, but the other components of Lancet will not normally need to be extended for most users.

Finally, a Launcher pulls together the Arguments and Command objects to launch the specified jobs on a particular platform. Currently, jobs can be run either locally with the Launcher, or with Grid Engine using the QLauncher. As the Launcher object accepts the other two core component types as arguments and is a fully declarative object (as are all Lancet components), a Launcher object fully specifies the intended parameter space, the command to execute, and the platform to execute it on.

### 3.1. Succinctly specifying a parameter space with lancet

**Figure 2:**
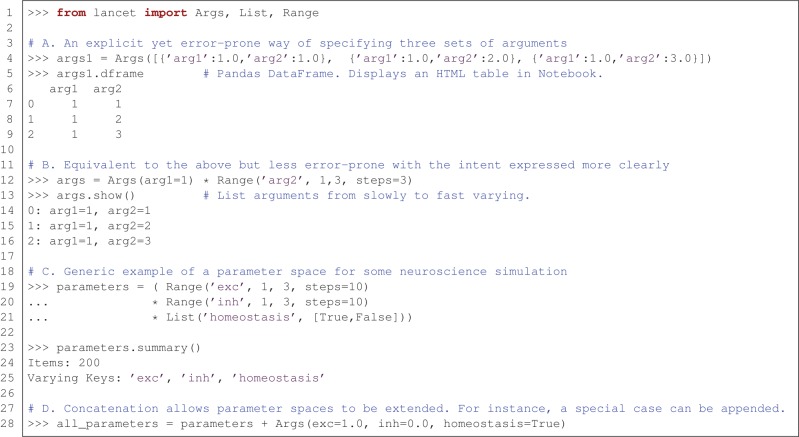
**Arguments express parameter spaces succinctly and declaratively. (A)** Example illustrating the most basic, most explicit use of the Args class to specify three sets of sequential arguments. **(B)** A more succinct and less error-prone way of specifying the same arguments. **(C)** An example expressing a parameter space for use with a hypothetical neural simulator. This parameter space covers a range of excitation and inhibition strengths, while toggling a homeostatic mechanism. **(D)** The concatenation operator allows arguments specified by Arguments objects to be sequenced, allowing special cases to be incrementally appended to a parameter space.

Figure 2 demonstrates some of the fundamental properties of all Arguments objects. These objects express parameter spaces that will result in many sets of parameter values to be passed to an external analysis tool or simulator, e.g., as command-line arguments. These are simple, compositional objects designed to express declarations of intent, independently of the other two types of Lancet component.

Part A of Figure 2 shows the most basic and explicit example of an Arguments definition, using an Args object to specify a static set of arguments. The list of dictionaries format is a verbose and completely flexible specification. However, this style of definition is neither succinct nor declarative, and therefore is not recommended unless absolutely necessary. Nonetheless, this constructor illustrates two key points: argument values are always paired with the corresponding argument name, and Lancet Args objects have a similar structure to the DataFrame objects used by the pandas data analysis library. As DataFrames accept an identical data format in the constructor, Lancet Args objects allow easy conversion to DataFrames via the dframe property (if the pandas library is available). This easy transition to the highly flexible pandas DataFrames data structure is a key part of enabling the agile workflow described in the next section. These objects are easy to create and automatically display themselves as HTML tables in the IPython Notebook environment.

Part B of Figure 2 expresses an identical parameter space using a more readable, less error-prone approach that clearly conveys the intended structure of the parameter space. In the explicit format shown in part A, the first argument 'arg1' remains constant with a value of 1.0 whereas the argument 'arg2' ranges over the numbers 1.0, 2.0 and 3.0. As a result, this parameter space is conveniently described as the Cartesian product of a constant argument for 'arg1' and a Range object that defining a range of values for 'arg2'.

The Cartesian product (also called the “cross product”) of different arguments is a natural way to specify parameter spaces, supported by Lancet Arguments via the multiplication operator. In imperative code, these appear as nested for loops where each parameter is iterated by one of the loops. The Cartesian product of Args(arg1=1) and the Range object is therefore a succinct way of declaring a parameter space with one argument kept constant as the second argument spans a range of values. Note that the Args object accepts arbitrary keyword arguments, allowing any constant values for named parameters to be easily declared.

Part C of Figure 2 shows a generic example of what a parameter space might look like in a simple, hypothetical neural simulation. A range of excitatory and inhibitory strengths is covered and a homeostatic mechanism is toggled on and off using the List declaration. Although simple, this object expresses 200 different argument sets (each leading to an independent simulation), as shown by the summary method.

Finally, in part D of Figure 2, the second compositional operator for Arguments objects is shown. The addition operator can concatenate (or sequence) Arguments objects together. The result is an object that first covers the parameter space of the first Arguments object before spanning the parameter space of the second Arguments object. This is a useful way to segment a parameter space in a piece-wise manner, allowing special cases to be easily added or the behavior at singularities to be investigated.

Using the Cartesian product and concatenation operations on the three basic Arguments objects, Args, List, and Range, many common parameter spaces can be expressed in a readily understood, compositional format. Arguments composed out of these basic objects have the property that the parameter space explored is known ahead of time, before jobs are executed. Although this is typical for many research tasks, Lancet also allows parameter spaces to be explored in an online fashion, where results returned by the jobs determine what portion of the parameter space is to be explored at the next step. Online parameter space exploration algorithms can be implemented in Lancet by subclassing DynamicArguments.

**Figure 3:**
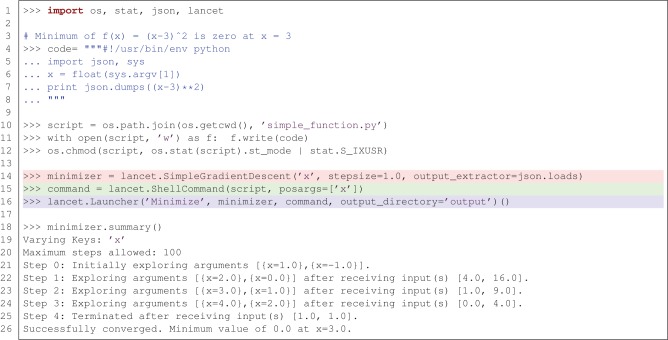
**Lancet allows dynamic exploration of parameter spaces using components of type DynamicArguments.** In this example, the minimum of *f* (*x*) = (*x* − 3)^2^ is found using SimpleGradientDescent, starting from *x* = 0 and terminating at the minimum where *x* = 3.

Figure 3 illustrates how Lancet can be used to dynamically explore a simple parameter space using the SimpleGradientDescent component. This instance of DynamicArguments is designed to demonstrate how a simple gradient descent algorithm operating on a single, scalar argument can operate in Lancet. In Figure 3, ShellCommand is used to run a short script that evaluates the function *f* (*x*) = (*x* − 3)^2^ on the input argument × when executed. SimpleGradientDescent then explores the local parameter space from the starting point *x* = 0 in steps of magnitude stepsize. Driven by the output of the script, SimpleGradientDescent descends the local gradient in × until it terminates at the local minimum, *x* = 3. In practice, well-established optimization procedures are likely to be more useful than this example class, such as those available in scipy.optimize, when trying to optimize parameter spaces that are not solvable analytically. Thus SimpleGradientDescent should be considered as one example of the types of DynamicArguments that can be implemented for advanced parameter space exploration procedures such as hill climbing or genetic algorithms.

In summary, the Arguments objects are declarative, composable objects that can vary from simple declarations of constant argument values to complex optimization procedures. In addition to the Arguments objects presented so far, Lancet offers FilePattern Arguments for matching filenames. The filenames found may then be used as arguments for a simulator, or used to specify a list of files for loading into the Python environment. There are also other more specialized Arguments objects such as Log, which allows previously explored parameter spaces to be loaded from the .log files saved by Lancet when running external tools.

### 3.2. Specifying how lancet supports your external tools

There are many different simulators and analysis tools used in computational neuroscience, each constantly being developed and updated. Some popular neural simulators include Brian (Goodman and Brette, 2008), Neuron (Hines and Carnevale, 1997), and NEST (Gewaltig and Diesmann, 2007), each of which uses different custom command-line interfaces. The most general approach to support such a wide range of tools is to treat them as external executables run on the command line. If a command-line specification is impractical or not supported by a particular tool, it is straightforward to write a Command that instead writes the specification for a run to a file to be read by the external program.

Even if you have the option of working exclusively with Python, such as for the Brian simulator, there can be clear advantages to writing your Python scripts as independent tools that can be invoked on the command line. Firstly, doing so ensures that independent runs are genuinely separate, sandboxing execution into separate processes to guarantee that independent jobs will not interact in unexpected ways. This requirement for process independence is explicit when running jobs on a cluster (for instance, when using Grid Engine). It is therefore useful to define a command-line interface to your Python scripts (perhaps using the argparse module) if you want code that can be executed both locally and in parallel on a cluster. Finally, defining a clear command-line interface can help document your code and allows useful standalone utilities to be pulled out of your code base.

When invoking tools with a standard command-line interface, Lancet supplies ShellCommand which can help avoid writing explicit interfacing code in many situations. For instance, ShellCommand is used to invoke the factor command in Figure 1. The ShellCommand is an instance of a Command that defines how Lancet can invoke an external tool via the command line. ShellCommand only supports communication via command-line arguments, but other Command classes may e.g., generate specification files appropriate to the chosen tool.

For interfacing with complex external software, users will often need to write a new Command subclass to extend Lancet's functionality for the new tool. Writing such a class is straightforward, as the subclass only needs to implement a constructor and a __call__ method. The __call__ method is supplied with arguments generated by an Arguments object in dictionary format (along with optional runtime information) and the Command must then return a list of strings suitable for Python's subprocess.Popen class. If the tool needs to load arguments from file, the Command may also save part of the parameter list specification to disk in an appropriate format before the command is executed. As described in the Discussion section, a special Command type could also be used to group small, lightweight jobs to avoid startup overhead.

Such interfacing code is designed to be simple, allowing the user to easily support new tools as required. These new components can then be supplied in a “Lancet extension” which may be bundled with the external software. For instance, the Topographica project (Bednar, 2009) offers a sophisticated Command subclass in a file named lancext.py. This component can invoke the simulator with a particular model file and defines Python analysis and measurement code for execution across a specified list of simulation times. Note that in this particular use case, although the Command passes the model file path to the command line, all parameters are specified on the command line rather than in the model file.

The lancext.py code is sufficiently flexible to support day-to-day exploratory work using the simulator, and was used throughout the development of the results in Stevens et al. (2013). The Command used is called RunBatchCommand, and is highlighted in green in Figure 5. The overall approach is general enough to be applicable to any simulator or tool, ranging from simple programs like factor, to complex neural simulators like Topographica, or even for running complex software outside the scope of computational neuroscience, such as time-consuming microprocessor simulations.

### 3.3. Specifying your chosen computational platform

The parameter space and the chosen tool are defined independently and do not interact until a platform is chosen by selecting a Launcher object. The purpose of a Launcher is to take an Arguments object declaring a parameter space and feed the instantiated arguments to the Command, which then passes the appropriate command specification back to the Launcher, which executes the tool on the appropriate platform. As all the components needed to launch jobs and generate data form the arguments of the Launcher, the printed representation (also known as the repr) of the Launcher captures a complete specification of how the output files are created.

As Lancet itself only uses cross-platform portions of the Python library, code that uses Lancet can work across operating systems (Linux, MacOS, Windows). One reason to subclass Command to support a given tool is to ensure appropriate command-line invocations are generated across different operating systems. Simple tools with a consistent format of command-line invocation can instead be safely launched with ShellCommand, on any operating system.

Lancet currently provides a basic Launcher class for running jobs locally, and a subclass QLauncher that launches jobs with Grid Engine. Although the jobs are launched in very different ways, both classes ensure that the output is organized consistently. This approach ensures that the rest of the researcher's code can be used as-is across all the available platforms. For instance, code that needs to locate output files can use the same approach regardless of whether the files were generated locally or on a cluster. This is an essential feature for an agile workflow: as your requirements grow, it is important to have the option to painlessly transition from readily accessible local computational resources to a high-throughput cluster that can run your jobs in parallel, and then back again for debugging.

Lancet's QLauncher component wraps the Grid Engine qsub command and has been extensively tested on an open-source variant of the original Grid Engine system (Son of Grid Engine, version 8.0.0e). QLauncher assumes only the basic options applicable across the various versions of Grid Engine (Sun/Oracle/Univa Grid Engine) and should be usable on any machine where a Grid Engine qsub command is available. More information about Grid Engine and the Son of Grid Engine project may be found at http://arc.liv.ac.uk/SGE/.

In addition to making the process of switching between platforms easy, Launchers help save important information alongside the output data that help ensure reproducibility and assist in later analysis. The .info file contains metadata which records important details requested from the version control system, the active Python and Lancet versions, operating system information and the complete representation of the source Launcher. The .log file contains an explicit list of all parameters used, allowing output to be quickly associated with the parameters used to generate it. This feature provides scientific provenance information for data analysis, which is crucial because the files output by a tool do not necessarily include the scientifically relevant parameters that were used to generate that data.

## 4. A realistic, agile, and evolvable workflow

Having introduced the general facilities offered by Lancet, we now examine how it can enable an agile and reproducible workflow using IPython Notebook. The use of external Python packages as appropriate is encouraged, and in particular the pandas library has proven very useful for analyzing data. To keep track of the code in the various Python scripts and IPython notebooks that appear as the workflow develops, it is also encouraged to keep a log of development by means of frequent code commits. Lancet works well together with distributed version control systems like Git and Mercurial, or with management and tracking tools tailored towards scientific use, such as Sumatra (Davison, 2012).

**Figure 4:**
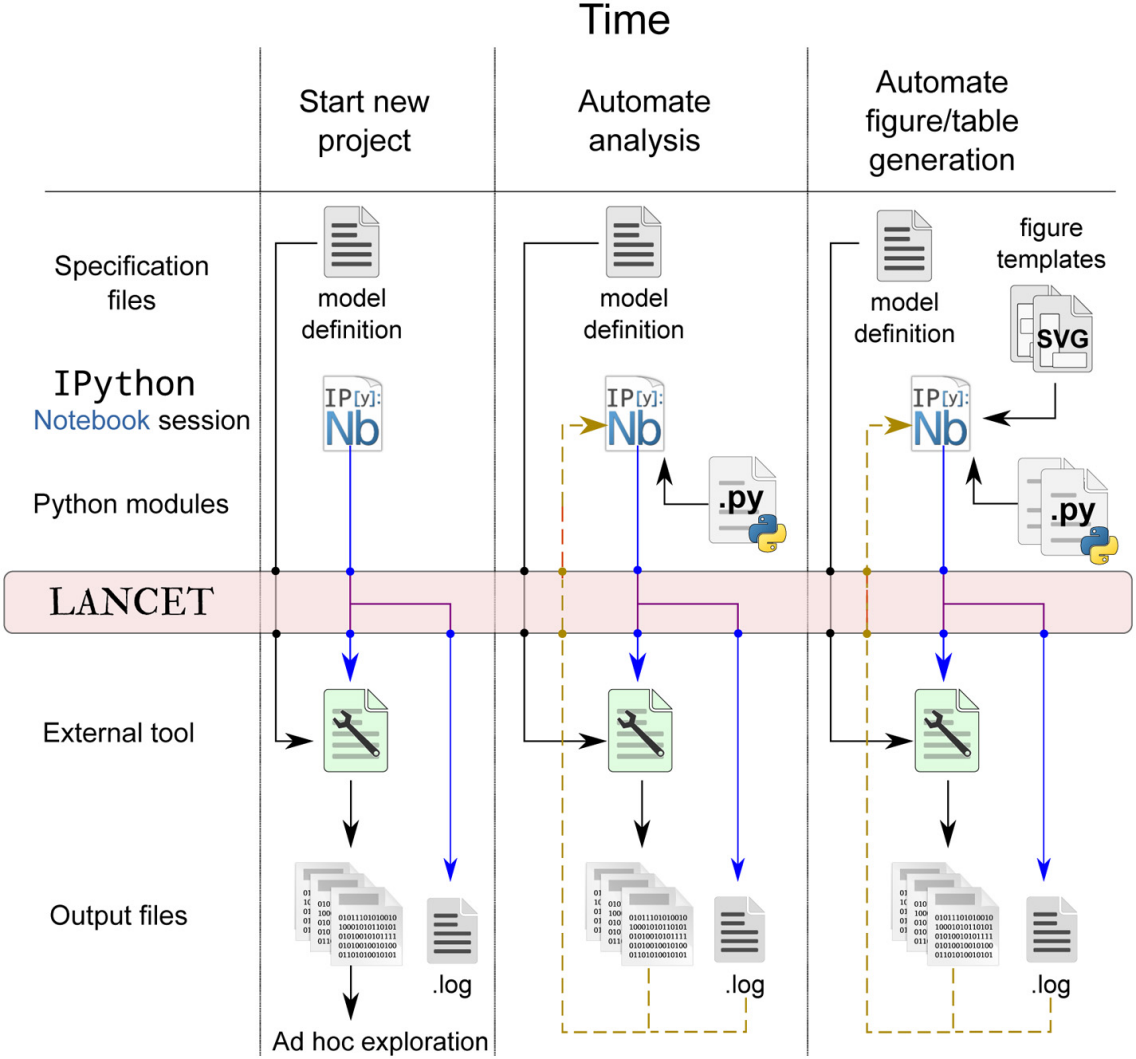
**Lancet captures a full declarative specification of the parameters, tools and platform employed, each time data is generated at every stage of the workflow.** Early in the project, the output files may be rapidly explored in an *ad hoc* way that does not need to be automated or reproducible, as illustrated in the first column. As the research project matures, more of the analysis and plotting procedure may be pulled back into IPython Notebook where it can be automated (middle column). Finally, as the research nears publication, SVG templates may be used to ease the automatic generation of publication figures, as shown in the last column.

Note that our proposed workflow using Lancet does not aim to be prescriptive or impose requirements on the user. It is our view that the researcher must primarily choose the tools that allows the most productive research possible. Our goal is therefore to make Lancet general and useful, allowing each researcher to organically develop their own workflow according to their own particular needs. By incorporating more Lancet components into your workflow over time, the code can become more succinct while increasing the overall level of automation and reproducibility. A schematic of how the workflow evolves over time is shown in Figure 4 and the stages of a typical research project using Lancet and IPython Notebook are now described:

1. An excellent way to start exploratory research is by creating a new IPython notebook. This offers an unconstrained environment where new ideas can be rapidly coded, tested and discarded as necessary. Using text and Markdown cells, notes can be interleaved with code to keep track of new ideas that relate either to scientific material or to coding. In this exploratory phase, the notebook is likely to be fairly disorganized and rapidly changing with many unrelated code snippets, outdated textual notes, HTML links, and other content (such as images) referencing external resources and documentation. Even so, even this early stage can be captured by committing the notebook to version control, preserving any progress made even though the user has not yet used any specific tool for reproducibility beyond the standard notebook.
2. Once a simulator or analysis tool has been chosen, small parameter spaces can be defined using the Arguments objects to be executed locally. If there is no Command available for the chosen tool, it is likely that ShellCommand will be sufficient to begin with. Otherwise, only a few lines of code are needed to subclass Command and satisfy the immediate requirements. At this stage, the output can be explored in an *ad hoc* manner, e.g., by inspecting files with a file manager or image viewer, as illustrated by the first column of Figure 4.
3. Lancet will store the repr (Python's term for an object's representation string) of the Launchers used along with the data in the .info files, maintaining a declarative record of how all the data was generated over time. As the project grows, it becomes crucial that version control is used to track notebook and code contents. A helper utility vcs_metadata is offered by Lancet that allows Git, Mercurial, or SVN version control information to be automatically stored in the .info files.
4. As the IPython Notebook is a very flexible environment for plotting and exploration, it quickly becomes worth writing small sections of Python code to automate away any *ad hoc* data inspection steps. It is also easy to load your data into the IPython notebook and rapidly generate plots with matplotlib. In particular, parameters associated with the loaded data can be brought into the notebook session by specifying a .log file to a Log Arguments object. This Log object may be used to re-run previously explored parameters, but also offers a convenient way to inspect and browse parameters previously logged by Lancet. By calling the dframe method of a Log object, a pandas DataFrame is generated that will present the logged parameters as an HTML table, offering a simple alternative to the web interface functionality offered by tools such as Sumatra. This stage is illustrated by the middle column of Figure 4.
5. Although small parameter spaces and local runs are often suitable initially when rapidly testing and debugging code, it is rare that this will prove sufficient for the whole project. As the code gets longer and more stable, it should be split out into Python modules to keep the notebook short and readable. As the code matures, parameter spaces tend to grow and simulation runs get longer and slower to obtain higher quality data sets. As the computational requirements increase, running simulations locally may become prohibitively slow, making it worth switching to a cluster if available. Lancet is designed to make such a transition painless:after switching Launcher for QLauncher and supplying a few basic settings appropriate to the cluster environment, the same code will immediately run in parallel on the cluster.
6. If a new Command class was implemented to support the external tool, this class may have matured to the stage where it is sufficiently general and flexible to become a reusable component, in which case it should also migrate to a separate file. By sharing this code with other Lancet users, the need to implement Commands will be alleviated in future as more and more tools are supported.
7. This particular stage of a research project may be quite prolonged, ending only when a particularly worthwhile avenue of research has been found. As the emphasis moves from exploration to publication, a particular subset of the code written is likely to become relevant. This code can be cleaned up and factored out into a Python module to keep the notebook manageable and to express the intentions of the developing paper clearly. Key plot types that are likely to become part of published figures may also be moved into a separate module.
8. In the final stages of developing a paper for submission, it can become cumbersome to generate complex, publication-quality figures using matplotlib alone. For this reason, to generate the final Figures in Stevens et al. (2013), a different approach was used—a small utility was written that allows SVG templates to be quickly authored in the Inkscape graphics editor. This utility then can then embed vector assets dynamically generated by Matplotlib to create the final, publication quality figure. At this stage, the notebook should embody a completely automated and reproducible workflow for published work, as illustrated by the final column of Figure 4 and demonstrated for Stevens et al. (2013).

The key characteristic of this proposed workflow is that although the final outcome is an IPython notebook that captures and automates all the steps needed to generate a published result, there is no stage where the researcher needs any motive other than a desire to increase productivity. Writing a new Command to interface with a new external tool (if such a class is not already available) may at first appear more trouble than writing a simple, *ad hoc* script such as a shell script, a Python script using subprocess, or a script in some other language such as Perl. But the key difference is that the initial Command is normally trivial, using a few lines of code to return a fixed list of strings to the command line.

Unlike *ad hoc* scripts that can rapidly become unmanageable, a new Command class remains maintainable as it becomes more general and useful, remaining viable across multiple research projects. Implementing such an object allows the same, clean declarative representation to be seamlessly used with either local simulations or when working on a cluster. A workflow that relies on scripting solutions to individual problems as they appear is likely to become unreadable over time, and is unlikely to be reused between projects. To illustrate, the RunBatchCommand and associated classes implemented for the Topographica simulator now offer significantly more functionality for batch simulations than was initially available with the simulator. Although the latest Topographica Lancet extension is still under 500 lines of code and documentation, it has helped make regular research work with this simulator much easier than before.

**Figure 5:**
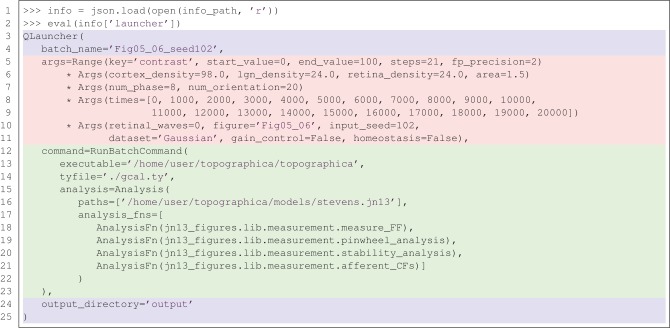
**A real example of recreating a launcher from the complete, declarative specification saved to the .info file.** The repr (the string representation) of the launcher is shown above, matching the corresponding string saved in the .info file. This example fully specifies 21 Topographica simulations used to generate Figures 5 and 6 from Stevens et al. (2013). Using a version control system also allows the state of the executed code (simulator, analysis, measurement code etc) to be restored based on the information stored in the .info file.

So far, the declarative, reproducible nature of Lancet objects has only been demonstrated with very simple examples. Figure 5 shows the full specification for a batch of Topographica simulations used in Stevens et al. (2013) in the form of a launcher repr. This newly created object can be run to regenerate the same data, without needing the notebook that originally launched it. The printed representation of the Launcher object shown in Figure 5 contains a real example of how the RunBatchCommand component is used in practice.

In this example, the .info file in one of the output directories is loaded using the json library and the contents of the Launcher key is evaluated. As the repr of a Launcher is always saved to the .info file and this repr is a complete, declarative object that is a valid Python expression, running eval(info['launcher']) creates a new Launcher with identical behavior to the original. This object is easily inspected and captures the full set of parameters, including the path to the simulator executable, the executed Topographica model file, and a list of analysis functions to be executed repeatedly over the course of each simulation run.

Calling this object without supplying any arguments in a cluster environment would relaunch the 21 Topographica simulations necessary to regenerate Figure 5 from Stevens et al. (2013). This code will reproduce identical results, as long as the Topographica simulator is working correctly. If the results change due to differences in the simulator code, the recorded version control information allows all the code to be restored to the same state as when the data was originally generated. Note that the code listing in Figure 5 is only one of the launchers needed to reproduce all the Figures in Stevens et al. (2013). In total, 842 simulation jobs were specified with Lancet to generate all the figures of the paper. Each job (simulation and analysis) takes over an hour to complete, so the full set of jobs takes several days to complete when running on a cluster, but the entire specification is still compact and human-readable.

## 5. Discussion

This paper has demonstrated a lightweight, flexible, and pragmatic approach to achieving scientific reproducibility without constraining innovation. There are many other approaches also available, ranging from just writing a complete Python script to automate all your tasks, to using a heavyweight workflow-automation system. These more ambitious workflow engines are in regular use by large commercial organizations and research groups in some fields (Freire et al., 2011), but are not currently common in computational neuroscience. Such workflow engines are typically designed to manage complex workflows with long pipelines, involving many different people. In contrast, the workflow presented here is designed to be minimalistic, suitable for small groups of researchers who wish to keep their research work flexible and do not want to embrace more complex and prescriptive workflow tools.

Our aim is to show that for a general class of exploratory research in Python, using IPython Notebook and Lancet together allows for an agile workflow that very naturally gradually becomes more reproducible and automated over time. The final result of this process is a set of IPython notebooks that fully reproduce published scientific results, without constraining the user at any stage of the process. Lancet deliberately does not prescribe any fixed way of doing research, and every component offered to the user should be evaluated on the basis of how well it improves immediate research efficiency.

As a historical note, each of the components of Lancet was originally developed to satisfy the needs of a real research project spanning multiple years, not simply to try to achieve reproducibility after the fact. In this project, many hundreds of simulations were executed locally using Lancet, and tens of thousands of jobs were launched on a cluster. But unlike the custom, *ad hoc* scripts that would normally be the result of such a project, Lancet was designed from the start to work just as well for completely different scientific domains, to ensure that the concepts and tools would be general and meaningful long into the future.

As a general tool, Lancet does not become any less relevant to research in computational neuroscience. To the contrary, having a general approach ensures that the essence of a workflow is valid over time as the underlying simulator tools come and go. The flexible and compositional nature of Lancet objects is suited to fast, exploratory research of interest to the computational neuroscience community using Python. Even though Lancet is newly available, it has already formed the basis for a complete scientific publication, made publicly available as an IPython notebook that automatically reproduces all the scientific results of the paper. This notebook allows all the code and results to be presented in a clear, automated way, and may be viewed and downloaded from the models/stevens.jn13 subdirectory of Topographica's GitHub repository.

For a tool that aims to be general, it is unsurprising that some functionality overlaps with other projects, given the many excellent third party libraries available for Python. For instance, there are several projects that offer sophisticated interfaces with Grid Engine, such as pythongrid and drmaa-python. IPython itself includes the IPython.parallel package which can help accelerate the pace of interactive work on a cluster. Some of the goals of Lancet's Arguments objects are shared by the parameters module of the NeuroTools package, which also allows parameter spaces to be defined. What distinguishes Lancet from these other libraries is that it offers all the tools needed to span an entire agile workflow with a collection of independent, declarative objects that work together.

Various workflow tools already exist with the computational neuroscientist in mind. VisTrails (Freire et al., 2014) is a scientific workflow and provenance system that integrates well with Python projects, taking a GUI-centric approach. The Mozaik framework (Antolík and Davison, 2013) is designed to encapsulate the workflows relevant to researchers who use spiking neural models. In contrast to these projects, Lancet is lightweight, with almost no dependencies, and is not tied to any particular set of simulator tools or workflows. Researchers exclusively using the appropriate spiking simulators may find Mozaik to be more specialized for their needs than Lancet, while Lancet is suitable for those who desire a more interactive workflow or need to use a broader class of tools or tools that are expected to change over time.

Projects like Sumatra (Davison 2012) take a far more general approach for achieving reproducibility, tailoring functionality offered by version controls to the needs of the scientist. In this way, Sumatra offers functionality that is orthogonal to Lancet, allowing both tools to be used successfully together. Lancet's approach aims for the middle of the spectrum between Sumatra and Mozaik, capturing declarative specifications within Python code that assists with automation and reproducibility without losing generality. Lancet is BSD-licensed and supports Python 3, and helps the researcher exploit well-established tools such as IPython Notebook and pandas in a way that makes day-to-day research easier and ultimately makes results more reproducible.

Lancet is also extremely extensible. The interface between Lancet objects has been deliberately kept simple, to allow new components to be added whenever required. The Command class allows Lancet to work with new external tools, invoking the tool appropriately for each set of arguments specified. In some situations, individual jobs may run quickly relative to the time for setup and initialization, making it inefficient for Lancet to span the parameter space directly. In such cases, Lancet can instruct the tool to cover the parameter range itself, with Lancet only specifying starting and stopping points (e.g., Args(start = 0, end = 5)). If necessary, the Command object could then use these values to build a range specification in a format the tool can use.

The process of executing jobs may also be customized to satisfy specific needs. For instance, there are currently two types of Launcher, one for running jobs locally and one for running jobs on Grid Engine. Other types of Launcher may be written to extend Lancet to new platforms. For instance, it should be very straightforward to write a Launcher that launches jobs over SSH, or one that allocates computational resources on demand with Amazon EC2. This new Launcher would then fit seamlessly into the other components offered by Lancet.

The Arguments objects are also designed to be extensible. Although the basic objects offered are already suitable for many research requirements, new Arguments objects can be written if desired. By building a new DynamicArguments component, Lancet can be used for more complex, online parameter space exploration, utilizing optimization techniques such as hill climbing or genetic algorithms. Currently, SimpleGradientDescent is the only such object supplied with Lancet, designed to demonstrate how more practical algorithms may be quickly implemented. It is hoped that the ability to employ optimization algorithms as necessary will extend the utility of Lancet and that by making use of mature, third party libraries, users will easily be able to rapidly implement the optimization procedures necessary to solve their problems.

Of course, it is important to remember that Lancet is just one small part of a toolset for achieving reproducibility. More-basic tools like Python, pandas, and matplotlib are crucial for making it practical to automate scientific tasks, which is a prerequisite for being able to capture the process for later playback. Distributed version control systems like Git and Mercurial make it easy to capture the state of anything that can be expressed in text. IPython Notebook and matplotlib make it feasible to explore and analyze results in a text-based way that can be captured by the VCS. Lancet simply helps tie these together with launching runs and collating the results, to fill in the missing pieces that allow the entire process to become reproducible in practice. In that way, it addresses the fundamental barrier to reproducibility, which is the large and extra investment of time and effort that would be needed to automate and preserve tasks once the research has been published.

Essentially, what Lancet offers are the missing utilities that make it easy to capture all the required steps within a single IPython notebook, from initial exploration to published results. Using Lancet you can quickly specify and launch jobs, keep output files consistently organized, switch from local execution to working on a cluster, record metadata and other key information together with your data, and load simulation output back into the notebook for analysis and plotting. By keeping everything under version control, the entire scientific process can then be captured, providing a flexible and agile yet reproducible research workflow.

The IPython notebooks that fully and automatically reproduce Stevens et al. (2013) are publicly available from the GitHub repository of the Topographica project (www.topographica.org) in the models/stevens.jn13 directory (https://github.com/ioam/topographica/tree/master/models/stevens.jn13). Lancet itself is freely available under a BSD license and may be downloaded from http://ioam.github.io/lancet/. Other examples of using Lancet are available at these Web sites.

### Conflict of interest statement

The authors declare that the research was conducted in the absence of any commercial or financial relationships that could be construed as a potential conflict of interest.
